# Preceding exercise and postprandial hypertriglyceridemia: effects on lymphocyte cell DNA damage and vascular inflammation

**DOI:** 10.1186/s12944-019-1071-y

**Published:** 2019-05-29

**Authors:** Malcolm Brown, Conor M. McClean, Gareth W. Davison, John C. W. Brown, Marie H. Murphy

**Affiliations:** 1 Sport & Exercise Sciences Research Institute, Ulster University, Jordanstown County Antrim, Northern Ireland BT37 0QB; 2 School of Nursing & Midwifery, Medical Biology Centre, Queen’s University Belfast, Belfast, Northern Ireland BT9 7BL

**Keywords:** Exercise, Postprandial hypertriglyceridemia, Oxidative stress, DNA, Inflammation

## Abstract

**Background:**

Exercise has proved effective in attenuating the unfavourable response normally associated with postprandial hypertriglyceridemia (PHTG) and accompanying oxidative stress. Yet, the acute effects of prior exercise and PHTG on DNA damage remains unknown. The purpose of this study was to examine if walking alters PHTG-induced oxidative damage and the interrelated inflammatory mechanisms.

**Methods:**

Twelve apparently healthy, recreationally active, male participants (22.4 ± 4.1 years; 179.2 ± 6 cm; 84.2 ± 14.7 kg; 51.3 ± 8.6 ml·kg^− 1^·min^− 1^) completed a randomised, crossover study consisting of two trials: (1) a high-fat meal alone (resting control) or (2) a high-fat meal immediately following 1 h of moderate exercise (65% maximal heart rate). Venous blood samples were collected at baseline, immediately post-exercise or rest, as well as at 2, 4 and 6 h post-meal. Biomarkers of oxidative damage (DNA single-strand breaks, lipid peroxidation and free radical metabolism) and inflammation were determined using conventional biochemistry techniques.

**Results:**

DNA damage, lipid peroxidation, free radical metabolism and triglycerides increased postprandially (main effect for time, *p* < 0.05), regardless of completing 1 h of preceding moderate intensity exercise. Plasma antioxidants (α-tocopherol and γ-tocopherol) also mobilised in response to the high-fat meal (main effect for time, *p* < 0.05), but no changes were detected for retinol-binding protein-4.

**Conclusion:**

The ingestion of a high fat meal induces postprandial oxidative stress, inflammation and a rise in DNA damage that remains unaltered by one hour of preceding exercise.

## Introduction

Metabolic disturbance following a high fat meal (HFM) has emerged as an independent risk factor for atherosclerosis [1]. This state, referred to as postprandial hypertriglyceridemia (PHTG), is characterized by an accumulation of triglyceride-rich lipoproteins (TRL) within the circulation [2]. With this greater abundance of metabolic substrate, cellular respiration becomes elevated, and as a consequence, reactive oxygen species (ROS) are generated [3]. Bae et al. (2001) [4] confirmed such a response after revealing PHTG-stimulated leukocyte superoxide (O_2_^•–^) production.

The primary reactive oxygen-derivative generated in cells is O_2_^•–^, and while it can react with, and damage molecular components, it also serves as a reducing agent, forming potent secondary radicals [5]. The interaction of O_2_^•–^ and nitric oxide (NO^•^) in the vasculature has been widely posited, yielding the potent oxidant peroxynitrite (ONOO^−^) and subsequent inhibition of endothelial function [6]. Further, such reactions can induce lipid peroxidation, protein damage and deplete antioxidants [7]. Many studies, including research from our own group, have reported that PHTG impairs vascular function, possibly via this or another oxidative stress mechanism [8, 9]. In addition to lipid and protein damage, ROS (namely secondary, injurious derivatives) have the capability of causing DNA single-stand breaks (SSB) and base oxidation [10]. It has been estimated that a single DNA structure is directly attacked 2 × 10^4^ times per day, the majority of which is attributed to free radicals [11]. Every aspect of the complex DNA structure is susceptible to oxidation, and reactive species have been directly implicated in more than 30 base modifications and 70 lesions [12]. With the observed increase in cellular respiration during the postprandial period, it is conceivable that SSB are exacerbated during these conditions. At this time, it has been reported that a single bout of exercise can damage DNA [13], but the effects of a HFM in this paradigm have been neglected.

Alongside this oxidative response, a HFM stimulates the secretion of several pro-inflammatory mediators, which further impacts the vasculature [14]. Given that both oxidative and inflammatory processes share common and overlapping signalling mechanisms, increased concentrations of one may reciprocate an increase in the other [15]. Nappo et al. [14] reported a sustained systemic increase in plasma cytokines (tumour necrosis factor (TNF)-α and interleukin (IL)-6) and adhesion molecules following a HFM. Similarly, Norata and colleagues [16] confirmed the expression of pro-inflammatory cytokines following an oral fat load in patients with hypertriglyceridemia. A HFM may also have the capacity to alter adipokine secretion [17] although data relating to the novel adipokine, retinol-binding protein-4 (RBP-4), is limited. RBP-4 may inhibit insulin signalling and provoke pro-inflammatory signalling in macrophages, meriting further investigation [18]. Taken together, the findings imply cumulative oxidative and inflammatory responses during the postprandial period. As such, PHTG is now of great biomedical interest, as many adults spend most of their waking day in this fed state [19]. With repeated and persistent periods of PHTG, individuals are continually exposed to an array of health impairing stimuli.

Several reviews advocate prior aerobic exercise to ameliorate some of the unfavourable effects of PHTG [20, 21]. A relatively recent meta-analysis showed prior exercise promotes a moderate reduction in postprandial triglycerides (TG) and incremental area under the curve TG [22]. Commonly, studies deploy prolonged bouts of exercise (90–120 min) to achieve such a response, with exercise volume postulated to influence the rate of appearance, or clearance, of TRL particles [23]. However, with shorter bouts of exercise (< 1 h) the evidence is contrasting [24, 25]. Such inconsistencies may be attributed to the differing exercise stimulus, composition of the test meal, or the meal timing in proximity to the exercise bout [22]. While moderate intensity exercise is a common feature of many exercise and postprandial studies, due to its influence in clearing lipoprotein remnants, recent studies have sought to clarify the influence of high intensity exercise (some intermittent in nature) on postprandial metabolism [22, 23]. The value of high intensity exercise is now widely recognized, especially in provoking a range of training adaptations [26]. However, for this current study we selected moderate intensity exercise to extend on the breadth of previous evidence, examining the oxidative and inflammatory milieu, while maintaining focus and continuity with the literature. Compared with high intensity exercise, moderate intensity exercise (i.e. brisk walking) is (arguably) more achievable for the general population and most likely avoids the potential confounding of exercise-induced oxidative stress, in addition to the anticipated postprandial oxidative stress following the meal ingestion.

Even though exercise and PHTG have received recent research attention, the precise impact of the postprandial oxidative stress and inflammatory milieu remains unclear [21]. Given that ROS are considered a main endogenous source of oxidative DNA damage [27], it is entirely conceivable that DNA could be altered as a result of HFM ingestion. Furthermore, the potential protective effects of exercise in this paradigm have been hitherto neglected. This study aims to address this evidence gap and investigate whether exercise, at an intensity suitable for most people, mitigates the oxidative and inflammatory effects induced by PHTG and how, if at all, they relate to DNA damage.

## Methods

### Participant characteristics

Following approval from the University Ethics committee, and in accordance with the Declaration of Helsinki (1964), twelve (*n* = 12) apparently healthy and recreationally active (approximately 2-h week^− 1^ exercise) male participants (22.4 ± 4.1 years; 179.2 ± 6 cm; 84.2 ± 14.7 kg; 51.3 ± 8.6 ml·kg^− 1^·min^− 1^) were recruited. Prior to commencement, all participants completed a health history questionnaire and provided informed consent, following disclosure of potential study risks and benefits. All participants were non-smokers and free from medication and antioxidant supplementation.

### Experimental design

Participants completed a randomised crossover study consisting of two trials: (1) ingestion of a high fat breakfast following one hour of rest (control) and (2) ingestion of a high-fat breakfast following one hour of moderate intensity walking exercise (immediately prior to the test meal). Participants were randomly assigned their initial condition, via random number generator, using Microsoft Excel. Following the HFM, participants were observed in the laboratory for 6 h. Each experimental trial was separated by 7 days and participants were asked to be as inactive as possible, limiting exertion to light walking only, as well as refraining from alcohol consumption, 24 h prior to testing. Dietary intake was recorded by each participant, using a food template, 24 h prior to testing and replicated for both conditions. Participants were tested following a standard 10 h overnight fast.

### Maximal oxygen consumption (V̇O_2max_)

Prior to experimental trials, participants completed an incremental running V̇O_2max_ test to exhaustion on a motorized treadmill (H-P Cosmos, Germany). Oxygen uptake was measured using a standard calibrated laboratory gas analysis system (Cosmed Quarkb^2^, Italy). Heart rate (Polar Electro, Finland) and perceived exertion (using the Borg Scale) were monitored continuously, and a valid V̇O_2max_ was confirmed using the following criteria (1) respiratory exchange ratio ≥ 1.15 (2) a clear plateau in mean oxygen uptake (< 2 ml· kg^− 1^· min^− 1^) and (3) a heart rate within 10 beats·min^− 1^ of age predicted maximum.

### Exercise protocol

Participants completed a single, 1 h bout of walking at 65% maximal heart rate. Participants remained at a steady walking pace for the entire duration. The treadmill speed was only adjusted to ensure each participant remained at the target heart rate zone (+/− 5 beats· min^− 1^). Heart rate was continuously monitored using an ECG short-range telemetry heart rate monitor (Polar Electro, Finland).

### High fat breakfast

The test meal consisted of commercially available bread, butter, cheese, mayonnaise, sausages and bacon, similar to previous work [8]. The content for each individual was calculated according to body mass, with each participant receiving 1.03 g of fat (68% energy content), 0.47 g of carbohydrate (16% energy content) and 0.59 g of protein (17% energy content) per kg body mass. The test meal was prepared by the investigator and consumed within 15 min. The meal represented an energy intake of 56.5 kJ per kg body mass (intake of 3958 kJ / 953 kcal for a 70 kg participant). Participants were limited to 500 ml of water while ingesting, which they drank ad libitum.

### Vascular measurements

Prior to measurement, participants rested in a supine position for 5 min. Pulse contour analysis, a measure of vascular function, was recorded using a Pulse Trace PCA2 monitor (Viasys Healthcare, UK). Systemic blood pressure (BP) was measured using an automated Omron MX2 Basic device (Surrey, UK). The mean of three tests was recorded. Vascular measures were obtained at baseline, post (exercise / rest) and at 2, 4 and 6 h post-meal.

### Biochemical analyses

### Venous blood sampling

Venous blood samples were obtained at baseline, immediately post (exercise or rest) at 2, 4 and 6 h post-meal using a 22-gauge intravenous cannula (Biovalve Safe, Vygon, UK) inserted into a prominent antecubital fossa vein. Blood was drawn into serum clot activator and K_3_EDTA vacutainers (Greiner Bio-One, Austria). Following collection, serum tubes were allowed to clot at room temperature for 10 min, while K_3_EDTA tubes were placed on ice. Blood tubes were centrifuged at 3500 rpm for 10 min at 4 °C (Hettich, Germany). Serum and plasma was extracted and stored at − 80 °C prior to biochemical analyses. Post-exercise blood samples were corrected for plasma volume shifts (see reference [8]).

### Analysis of blood samples

### DNA strand breaks

Lymphocytes were isolated using Histopaque®-1077 (3 mL) (Sigma-Aldrich, USA) and centrifuged at 3500 rpm for 30 min at 4 °C. The mononuclear layer was suspended in phosphate buffered saline (PBS) (Oxoid Ltd., Hampshire, UK) and centrifuged at 2600 rpm for 10 min at 4 °C (three washes). Isolated lymphocytes were slowly frozen to − 80 °C in a stable freezing medium (800 μL Roswell Park Memorial Institute medium, 100 μL Foetal Bovine Serum and 100 μL Dimethyl Sulfoxide (DMSO) (Sigma-Aldrich, USA). In preparation for analysis, microscope slides were pre-coated with 1% normal melting point agarose. The cell pellet was rapidly thawed using PBS (10 mL) centrifuged and re-suspended in 1 mL of PBS. Thereafter, 50 μL of the cell suspension was mixed with 150 μL of 1% low melting point agarose and applied to a pre-coated slide. The gel solidified under a 20 × 20 mm coverslip at 4 °C. The coverslip was gently removed and the slides were placed in a lysis buffer (2.5 M NaCl, 0.1 M EDTA, 10 mM Tris and 1% Triton X-100 (added immediately prior to use), pH 10) for 1 h at 4 °C. The slides were placed horizontally in an electrophoresis unit containing electrophoresis buffer (0.3 M NaOH, 1 mM EDTA, pH 13) for 20 min at 4 °C. Electrophoresis (25 V, 300 mA, 1.1 V/cm) was performed for 25 min at 4 °C. Slides were transferred to a staining jar and neutralized with PBS and distilled H_2_O at 4 °C for 10 mins each. Next, slides were immersed in SYBRGold for 40 min at 4 °C. Upon removal, the slides were washed twice and allowed to dry vertically in the dark. The gels were hydrated, covered and 50 random cells from each slide were analysed with the Comet Assay IV software (version 4.3, Perceptive Instruments Ltd., UK) at a magnification of 400 X using a fluorescent microscope (Olympus BX41, UK) and illumination system (Olympus U-HGLGPS, UK). The intensity of the tail length (%) was selected to quantify DNA damage.

### Positive DNA control slides

Resting human lymphocytes were used as positive DNA controls prior to each assay. 50 μL of cells were incubated with double dilutions of hydrogen peroxide (H_2_O_2_) (20, 40 and 80 μL of a 3% solution) for 20 min, and analysed as previously stated.

### Measurement of lipid hydroperoxides (LOOH)

Lipid hydroperoxides were measured in serum using the ferrous iron/xylenol orange (FOX) assay. The reagent was prepared using 25 mM.L^− 1^ of sulphuric acid (H_2_SO_4_), 250 μM.L^− 1^ of ammonium ferrous sulphate, 100 μM.L^− 1^ of sorbitol and 100 μM.L^− 1^ of xylenol orange in distilled water. Next, 90 μL of serum was mixed with 900 μL of reagent, incubated at room temperature for 30 min in darkness. Absorbance was measured using a UV spectrophotometer at 560 nm against a standard curve (Mason Technologies, Ireland).

### Electron paramagnetic resonance (EPR) spectroscopic analysis

The ascorbyl radical (Asc^•^) was measured in plasma using EPR spectroscopy. To start, 1 mL of plasma was mixed thoroughly with 1 mL of dimethyl sulfoxide (DMSO) in a glass test tube and 1 mL of the final solution was drawn into a sterile syringe and flushed into the analyser cavity. All samples were analysed at room temperature using a calibrated Bruker EMX series X-band EPR spectrometer (Bruker, Germany). The parameter conditions were set as follows: frequency (9.785 GHz); microwave power (20 mW); modulation frequency (100 kHz) and modulation amplitude (1.194 G) for three sweeps. Spectral parameters were obtained using commercially available software (Bruker Win EPR System, Version 3.2) and filtered identically. The relative concentration of the ascorbyl radical was determined by signal intensity.

### Determination of lipid soluble antioxidants

Endogenous lipid soluble antioxidants were measured using simultaneous high performance liquid chromatography (HPLC). Plasma samples were measured using the testing parameters outlined previously [28] for α-tocopherol, γ-tocopherol, retinol, lycopene, α-carotene and β-carotene at changing wavelengths of 292 nm, 325 nm and 450 nm. Results were interpreted by Empower software (Version 2, Waters Corp, USA).

### Retinol-binding protein-4

Serum samples were assayed for retinol-binding protein-4 using a Quantikine® sandwich enzyme-linked immunosorbent assay (ELISA) (R & D Systems, USA). Once prepared, the well-plate was read immediately at 450 nm and concentration was determined using a 4-parameter logistic curve fitting algorithm (ReadFit Pro 2014, Hitachi Solutions).

### Erythrocyte sedimentation rate (ESR)

Immediately following the collection of whole EDTA blood, 2 ml was transferred to a Westergren-Katz tube and placed vertically on a rack for 1 h at room temperature. The rate of erythrocyte aggregation (mm/hr) provides an indirect estimation of the presence of inflammation.

### Metabolic markers

Serum samples were assayed for triglycerides, glucose and total cholesterol (TChol) using enzyme assay kits (Roche Diagnostics, Germany) at the Ulster Hospital, Belfast. All analyses were conducted using a Roche/Hitachi Cobas c 701/702 system at wavelengths of 340 nm and 505 nm.

### Statistical analysis

Statistical analysis was performed using SPSS version 22 (IBM, Hampshire, UK). Data were analysed using a two-way repeated measures analysis of variance (ANOVA) with one between (trial) and one within (time) subject factor. For significant interaction effects, within subject factors were further analysed using a Bonferroni-corrected paired samples t-test. Between subject differences were analysed by one-way ANOVA with a posteriori Tukey Honestly Significant Difference (HSD) test. In the event of a main effect for time, data was pooled (exercise and control combined) and paired sample t-tests were performed. Summary measures of the triglyceride response to the meal ingestion were calculated using the area under the curve (AUC) and incremental area under the curve (iAUC) using the trapezoidal rule. Differences between the exercise and control trial were examined using the Student’s *t* test*.* The alpha level was set at *p* < 0.05. A prospective power calculation, factoring critical difference, was performed. Retrospective power calculations were carried out using SPSS. All data within is expressed as mean ± S.D. unless otherwise stated.

## Results

### DNA single-strand breaks

**Fig. 1:**
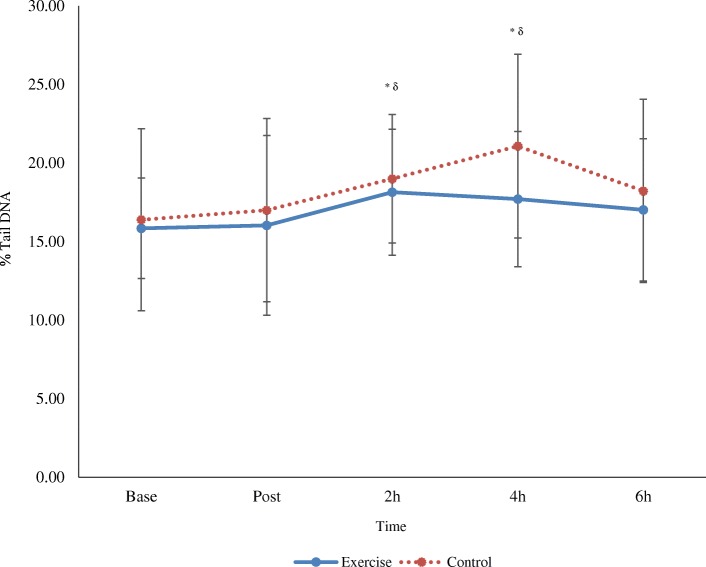
DNA damage over time following a high fat meal and preceding exercise (mean ± SD; *n* 12). Main effect for time at 2 h and 4 h post-meal versus baseline (^*^) and post (^δ^) (*p* < 0.05; pooled exercise and control group data)

**Fig. 2:**
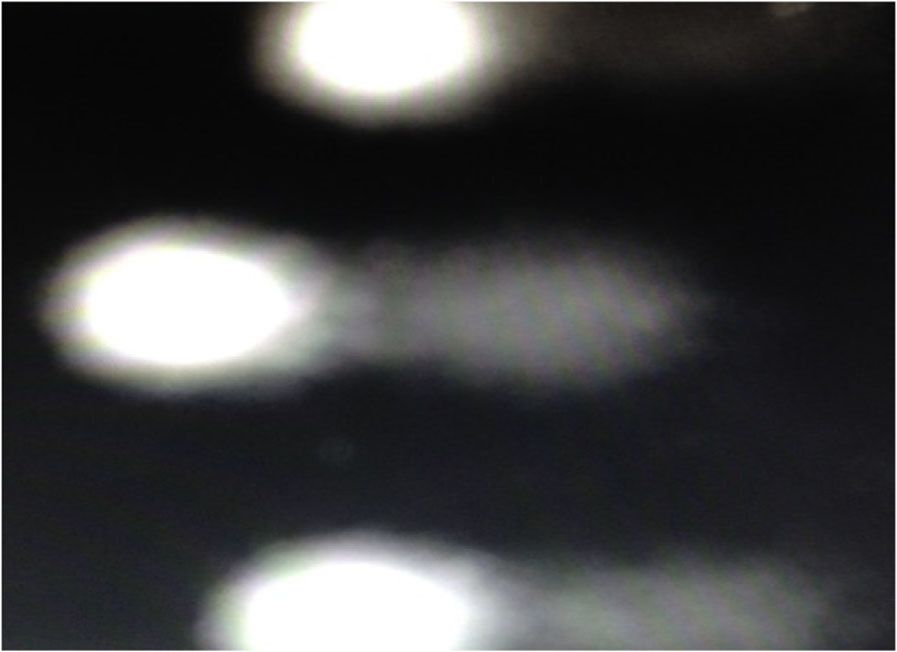
Representative image for the comet assay (obtained from a control group DNA slide at 2 h post-meal; median % tail intensity = 24.09)

Analysis of DNA damage revealed a main effect for time (*p* < 0.05) but no differences between conditions (time x trial interaction, *p* > 0.05). Further analysis of pooled data (both exercise and control conditions) indicates DNA strand breaks increased at 2 h and 4 h post-meal compared to baseline (p < 0.05) (Fig. 1). A representative image of the DNA analysis is provided in Fig. 2. A main effect for time was also detected for tail moment at 2 h and 4 h, compared to baseline and post (Table 1).

**Table 1:** Effects of exercise and the high fat test meal on additional comet assay parameters over time (mean ± SD, *n* 12)

	Trial	Baseline	Post	2 h Post-HFM	4 h Post-HFM	6 h Post-HFM
% Head DNA	Exercise	81.61 ± 12.9	82.15 ± 10	78.48 ± 14	82.26 ± 8.3	79.60 ± 12.5
	Control	82.67 ± 10.65	80.20 ± 10.98	80.83 ± 5.29	79.17 ± 6.78	83.65 ± 6.09
Tail length	Exercise	345 ± 110	340 ± 132	386 ± 141	350 ± 108	369 ± 119
	Control	361 ± 118	387 ± 119	397 ± 109	397 ± 135	341 ± 80
Tail moment	Exercise	22.14 ± 6	20.92 ± 6.92	26.77 ± 8.09^*#^	26.69 ± 8.47^*#^	25.66 ± 8.78
	Control	22.76 ± 8.62	25.71 ± 8.79	28.87 ± 7.18^*#^	29.34 ± 8.83^*#^	23.70 ± 8.79

Main effect for time for tail moment at 2 h and 4 h versus baseline (^*^) and post (^#^) (p < 0.05; pooled exercise and control data)

### Lipid hydroperoxides

**Fig. 3:**
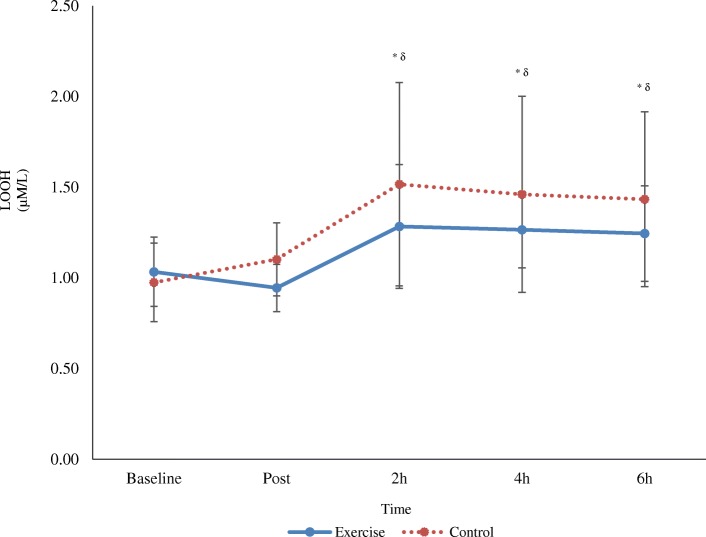
Effects of exercise followed by a high fat meal on lipid hydroperoxides (LOOH) over time (mean ± SD; *n* 12). Main effect for time at 2 h, 4 h and 6 h post-meal compared to baseline (^*^) and post (^δ^) (*p* < 0.05; pooled exercise and control group data)

There was no change in lipid hydroperoxide concentration between conditions over time (time x trial interaction, *p* > 0.05). There was, however, a main effect for time (*p* < 0.05) with pooled data demonstrating an increase from baseline and post at 2 h, 4 h and 6 h post-meal (p < 0.05) (Fig. 3).

### EPR spectroscopy

There was no time x trial interaction effect detected for the ascorbyl radical (*p* > 0.05). A main effect for time was revealed (*p* < 0.05), and further analysis of pooled data suggests the ascorbyl radical increased from 2 h post-meal at 4 h and 6 h (*p* < 0.05) (Table 2).

**Table 2:** Effects of exercise and high fat meal (HFM) on ascorbyl radical generation and lipid soluble antioxidants over time (mean ± SD, *n* 12)

	Trial	Baseline	Post	2 h Post-HFM	4 h Post-HFM	6 h Post-HFM
Asc^•^ AU	Exercise	− 836 ± 192	− 866 ± 226	− 868 ± 237	− 819 ± 210	− 778 ± 219^*δ^
	Control	− 838 ± 170	− 837 ± 169	− 832 ± 147	− 803 ± 116	− 779 ± 130^*δ^
γ-tocopherol (μΜ/L)	Exercise	0.94 ± 0.38	0.91 ± 0.37	1.00 ± 0.42^*δ^	1.13 ± 0.42^*δ^	1.33 ± 0.53^*δ^
	Control	0.92 ± 0.38	0.92 ± 0.40	1.00 ± 0.40^*δ^	1.19 ± 0.43^*δ^	1.40 ± 0.54^*δ^
α-tocopherol (μΜ/L)	Exercise	19.08 ± 3.62	19.80 ± 3.59^*^	19.86 ± 3.64^*^	20.21 ± 3.38^*δ^	20.85 ± 3.90^*δ^
	Control	18.14 ± 2.78	19.25 ± 3.57^*^	19.28 ± 3.50^*^	20.72 ± 4.28^*δ^	21.37 ± 4.30^*δ^
Retinol (μΜ/L)	Exercise	1.52 ± 0.42	1.84 ± 0.57	1.72 ± 0.70	1.62 ± 0.43	1.78 ± 0.76
	Control	1.63 ± 0.49	1.80 ± 0.45	1.71 ± 0.50	1.89 ± 0.78	1.86 ± 0.50
Lycopene (μΜ/L)	Exercise	0.67 ± 0.37	0.68 ± 0.37	0.61 ± 0.24	0.66 ± 0.36	0.60 ± 0.29
	Control	0.59 ± 0.20	0.63 ± 0.23	0.57 ± 0.12	0.60 ± 0.20	0.55 ± 0.12
α-carotene (μΜ/L)	Exercise	0.03 ± 0.01	0.04 ± 0.01	0.03 ± 0.02	0.03 ± 0.02	0.03 ± 0.01
	Control	0.04 ± 0.02	0.04 ± 0.02	0.03 ± 0.02	0.04 ± 0.02	0.04 ± 0.02
β-carotene (μΜ/L)	Exercise	0.21 ± 0.06	0.23 ± 0.07	0.23 ± 0.07	0.22 ± 0.07	0.22 ± 0.06
	Control	0.24 ± 0.08	0.25 ± 0.10	0.23 ± 0.10	0.24 ± 0.11	0.24 ± 0.12

^*^ Main effect for time compared to baseline (*p* < 0.05; pooled exercise and control group data). ^δ^ Main effect for time versus post (*p* < 0.05; pooled exercise and control data). *Asc*^*•*^*,* Ascorbyl radical

### Lipid soluble antioxidants

No within or between trial differences in lipid soluble antioxidants (retinol, lycopene, α-carotene and β-carotene) were detected over time (time x trial interaction, *p* > 0.05). A main effect for time was detected for γ-tocopherol (p < 0.05), and further analysis of pooled data shows an increase at 2 h, 4 h and 6 h post-meal (*p* < 0.05). A main effect for time was also identified for α-tocopherol with pooled data indicating that this metabolite increased immediately post-trial (*p* < 0.05), and again at 2 h, 4 h and 6 h post-meal (p < 0.05) versus baseline (Table 2).

### Retinol-binding protein-4

No changes were detected in retinol-binding protein-4, either within or between conditions (time x trial interaction, *p* > 0.05).

### Erythrocyte sedimentation rate (ESR)

There was no change in erythrocyte sedimentation rate between conditions (time x trial interaction, *p* > 0.05). A main effect for time was detected (*p* < 0.05), and further scrutiny indicates ESR increased from baseline at post-exercise/rest, 2 h and 4 h post-meal (p < 0.05).

### Metabolic markers

There was no change in triglycerides between conditions over time (time x trial interaction, *p* > 0.05). A main effect for time was observed (p < 0.05) and further analysis of pooled data indicates that the high fat meal caused an increase at 2 h, 4 h and 6 h versus immediately pre-ingestion (i.e. immediately post exercise and rest) (*p* < 0.05). The AUC for postprandial triglycerides showed no differences between the exercise and control trials (10.41 ± 5.02 mmol·l·6 h^− 1^ vs. 12.08 ± 7.46 mmol·l·6 h^− 1^; *p* > 0.05), respectively. Likewise, the iAUC for postprandial triglycerides showed no differences between the exercise and control trials (2.77 ± 1.29 mmol·l·6 h^− 1^ vs. 3.71 ± 2.70 mmol·l·6 h^− 1^; p > 0.05), respectively. Analysis of glucose concentrations revealed no significant change between trial over time (time x trial interaction, *p* > 0.05) but instead a main effect for time was seen (*p* < 0.05). Pooled data indicates that glucose decreased immediately following one hour of exercise and rest, and remained suppressed until 6 h post-meal. In addition, a time x trial interaction effect (*p* < 0.05) and main effect for time were observed for total cholesterol (p < 0.05). This significant change accounted for an increase within the control trial from baseline at 6 h (p < 0.05) (Table 3).

**Table 3:** Transient effect of exercise and a high fat meal (HFM) on metabolic markers over time (mean ± SD; *n* 12)

	Trial	Baseline	Post	2 h Post-HFM	4 h Post-HFM	6 h Post-HFM
TG (mmol/L)	Exercise	1.13 ± 0.62	1.06 ± 0.51	1.69 ± 0.83^*δ^	1.70 ± 0.80^*δ^	1.47 ± 0.87^*δ^
	Control	1.13 ± 0.67	1.16 ± 0.74	2.06 ± 1.17^*δ^	1.99 ± 1.39^*δ^	1.76 ± 1.27^*δ^
Glucose (mmol/L)	Exercise	4.82 ± 0.44	4.83 ± 0.37^*^	4.33 ± 0.30^*^	4.65 ± 0.23^*^	4.62 ± 0.16^*^
	Control	4.96 ± 0.41	4.68 ± 0.51^*^	4.32 ± 0.61^*^	4.59 ± 0.31^*^	4.67 ± 0.23^*^
TChol (mmol/L)	Exercise	4.30 ± 0.73	4.26 ± 0.74	4.37 ± 0.77	4.39 ± 0.66	4.38 ± 0.71
	Control	4.12 ± 0.68	4.21 ± 0.61	4.31 ± 0.82	4.29 ± 0.74	4.42 ± 0.71^¥^

^*^ Main effect for time versus baseline (*p* < 0.05; pooled exercise and control group data). ^δ^ Main effect for time versus post (*p* < 0.05; pooled exercise and control data). ^¥^ *p* < 0.05 *time x group interaction* versus baseline. *TG,* triglycerides; *TChol,* total cholesterol

### Vascular measures

There was no effect of exercise (time x trial interaction) on either systolic or diastolic blood pressure or on pulse contour analysis stiffness and reflection index (p > 0.05), but a main effect for time exists for all four variables (p < 0.05). Pooled data suggests systolic blood pressure increased following the high fat meal (p < 0.05), whereas diastolic blood pressure, the stiffness index, and reflection index were lower following the test meal (Table 4).

**Table 4:** Effects of prior exercise and a high fat meal (HFM) on vascular function over time (mean ± SD; *n* 12)

	Trial	Baseline	Post	2 h Post-HFM	4 h Post-HFM	6 h Post-HFM
Systolic BP (mmHg)	Exercise	126 ± 10	126 ± 10	129 ± 9^*^	126 ± 7	130 ± 6^*^
	Control	127 ± 7	127 ± 7	131 ± 7^*^	131 ± 11	132 ± 6^*^
Diastolic BP (mmHg)	Exercise	68 ± 6	70 ± 5	64 ± 7^*^	63 ± 5^*^	67 ± 6
	Control	68 ± 5	71 ± 6	65 ± 5^*^	66 ± 6^*^	69 ± 6
Arterial Stiffness (m/s)	Exercise	6.4 ± 0.7	6.2 ± 0.4	5.9 ± 0.5	5.9 ± 0.7^*^	5.8 ± 0.4^*^
	Control	6.1 ± 0.7	6.1 ± 0.6	6.2 ± 0.7	5.9 ± 0.6^*^	5.9 ± 0.5^*^
Reflection Index (%)	Exercise	68 ± 10	65 ± 11	60 ± 11^*^	61 ± 9	61 ± 11
	Control	71 ± 10	75 ± 12	69 ± 9^*^	68 ± 10	69 ± 8

^*^ Main effect for time compared to baseline (*p* < 0.05; pooled exercise and control group data). *BP,* blood pressure

## Discussion

The purpose of this study was to examine the transient effects of preceding moderate intensity walking and PHTG on biomarkers of inflammation and oxidative stress during the postprandial period. This study is the first to show DNA damage after a HFM, accompanied by an increase in other markers of oxidative stress, which all remained elevated following a bout of exercise. Moreover, PHTG promoted an increase in the activity of selective lipid-soluble antioxidants and markers of systemic inflammation, but no changes were observed in the novel adipokine, RBP4.

A main finding of this study is increased DNA damage at 2 and 4 h postprandially in both trials. The percentage tail DNA was selected as it provides a sensitive measure of low level DNA damage [29]. The mechanisms for this observed increase in DNA damage remains somewhat unclear, although a plausible candidate is increased postprandial ROS production and an associated rise in neutrophil activation [30]. In agreement, Van Oostrom and colleagues [31] reported increased postprandial neutrophil activation and a concomitant elevation in oxidative stress. Similarly, Alipour et al. [32] confirmed enhanced neutrophil and monocyte activation following PHTG. Perhaps the stimulation of NADPH oxidase (Nox) contributes to DNA damage, given it readily reduces O_2_ to produce O_2_^•–^ [33]. Nox enzymes are activated by several metabolic factors and, as such, TRL are capable of inducing leukocyte activation [34]. Neutrophils and monocytes also have the ability to uptake lipoprotein remnants and bind postprandial fatty acids, opening the possibility of direct activation by chylomicrons and their remnants [32, 34]. Alternatively, the rise in postprandial ROS may be attributed to the greater availability of metabolic substrate leading to an excessive production of NAD, increased mitochondrial proton gradient and subsequent transfer of electrons to O_2_ [1]. While the primary free radicals generated (O_2_^•–^ and NO^•^) do not appear to react at significant rates with DNA, O_2_^•–^ in particular, acts a precursor for the potent ^•^OH (hydroxyl) radical, that can readily promote DNA strand breaks and base modification [11]. ^•^OH can also directly react with purines creating an array of secondary free radicals (e.g. addition of ^•^OH to purines can generate adduct radicals with redox potential) [27, 35]. In previous reports, exercise has proved effective in mitigating intracellular ROS production following PHTG [36], yet this was not evident within the current trial and may be dependent upon meal timing, meal substrate composition, or the volume of exercise, thus allowing oxidative damage to persist. Perhaps a bout of high intensity exercise, in this recreationally active population, may have proved efficacious in reducing/preventing DNA damage. Nevertheless, higher intensity exercise may present further mechanistic complexities and disruptions to redox homeostasis; it is often documented for increasing ROS production, which could further accentuate the oxidative damage induced by the meal - consistent with the theory of hormesis [37].

As this is the first study to report postprandial DNA damage, further work is required to explore the significance, if any, of such findings. Nonetheless, free radical-induced DNA damage can have deleterious consequences to normal cell function and prompts a state of pathogenicity. Of the purines, guanine is particularly vulnerable to oxidation due to its low redox potential, with 8-hydroxyguanine (8-oxoG) the most abundant product of DNA damage [38]. This mutagenic base lesion challenges DNA glycosylase repair, and ineffective repair gives rise to base transversion mutations, that are common in a state of pathogenesis [39]. Mutations to either the DNA helical structure or base pairs, if uncorrected, likely lead to the manifestation and progression of disease [40]. Indeed, this supposition is purely speculative at this moment in time, as we did not quantify DNA repair or sequence changes in this investigation.

The current study, in agreement with prior research, also showed a HFM has the propensity to increase lipid peroxidation at 2, 4 and 6 h post-meal. We have previously shown that lipid hydroperoxides increase following PHTG, preceded by 1 h of moderate intensity exercise [41]. This process of lipid peroxidation is mediated by ROS where carbon-centred lipid radicals, often combine with molecular O_2_ to form lipid peroxyl (LOO^•^) radicals [42]. A chain reaction of repeated hydrogen abstraction and radical formation commences, and lipid hydroperoxides are generated once LOO^•^ reacts with hydrogen atoms [43]. The increased response observed may be associated with the accumulation of TRL and their downstream effects on free radical production and oxidative stress. α-tocopherol is an important chain breaking antioxidant that reduces LOO^•^ and thereby blunts the propagation phase. In doing so, a stable α-tocopheryl radical is generated and recycled during the oxidation of ascorbate to yield the ascorbyl radical [44]. In the current study, the ascorbyl radical increased at 6 h post-meal suggesting ongoing increased free radical production. To complement these findings, the mobilisation of selected lipid-soluble antioxidants (γ-tocopherol and α-tocopherol) increased. Perhaps the systemic rise in certain lipid-soluble antioxidants occurred as a scavenging response to PHTG-induced oxidative stress or indeed may have occurred as a result of exercise-induced adipose tissue lipolysis [45].

ESR, an indirect biomarker of inflammation, increased immediately post and at 2 and 4 h post-meal. As ESR is a non-specific measure, determining the inflammatory mediators present in venous circulation is nearly impossible, though given the oxidative response, they are likely pro-inflammatory in nature. In a recent review, Herieka and Erridge [46] reported cytokines and soluble adhesion molecules were not consistently raised following a HFM but pro-inflammatory leukocyte surface markers, mRNA and proteins were elevated in almost all studies in which they were measured. While few studies have examined ESR within this context, several have analysed specific markers of inflammation. Nappo and colleagues [14] report a sustained systemic increase in plasma concentrations of TNF-α, IL-6, intracellular adhesion molecule (ICAM)-1 and vascular cell adhesion molecule (VCAM)-1 following a HFM in healthy participants. Interestingly, this response was blunted with the ingestion of an antioxidant cocktail highlighting again the potential interaction effect between the two biological processes [14]. Similarly, Brandauer and colleagues [47] report increased IL-6 and IL-8 following a HFM and this was accentuated by prior moderate intensity exercise. More recently, in a similarly designed study, Teeman et al. [48] reported preceding moderate intensity walking could not mitigate the inflammatory response, stimulated by a HFM. Though preceding exercise, the previous day, may influence inflammatory signalling (AMPK and NF-κB) in peripheral blood mononuclear cells, suggesting exercise may stimulate anti-inflammatory mechanisms over time [49]. Like Brandauer and colleagues [47], the adipokine, RBP4 was measured, as it has been proposed as a key potential mediator of postprandial inflammation [47] and oxidative stress [50]. No changes were detected across experimental conditions and this is consistent with the findings of Brandauer et al. [47]. This data suggests that while RBP4 does not change over time, inflammation appears to persist highlighting once again the role of PHTG in endothelial activation. Further analysis to elucidate a possible role for RBP4 as a mediator of postprandial inflammation and oxidative stress is still warranted given that many of the existing studies, including our own, are somewhat limited by the analytical approaches to quantify RBP4 and a lack of controlling covariates for this novel adipokine [50].

Following ingestion of the HFM, TG increased at 2, 4 and 6 h across both experimental conditions. In agreement, similar studies have reported both moderate and high intensity exercise did not mitigate postprandial TG [48, 51]. In contrast, Katsanos et al. [52] reported 90 min of moderate exercise, 1 h prior to the administration of a HFM, reduced PHTG over time. We suspect the 50% greater time spent exercising augmented energy expenditure in the Katsanos study, accounting for the differences in triglycerides post-meal with our own findings. More recently, Kashiwabara et al. [53] reported continuous and intermittent walking decreased TG area under the curve to a similar extent, during the postprandial phase. Numerous methodological considerations may account for the observed disparity including energy expended and the posited subsequent upregulation of lipoprotein lipase (LPL) [20]. Nevertheless, there is now evidence to suggest that this does not necessarily need to change for concomitant reductions in TG [54]. Alternatively, the fat content of the test meal in the current study may have simply overwhelmed metabolic capacity for TG clearance and/or storage in our volunteers allowing a rise in TG. Humans can typically manage a dietary fat load of ≤15 g, with fat concentrations between 30 and 50 g sufficiently challenging triglyceride clearance capacity, prompting postprandial hypertriglyceridemia in a dose-dependent fashion. Yet, the response to meals with a fat content beyond 80 g is no longer dose dependent [21]. It’s our belief that such a large fat content overwhelms metabolic capacity for triglyceride clearance, prompting a rise in lipoprotein remnants, inflammation and thus enhanced free radical production and potentially disruptions to redox homeostasis. Furthermore, total cholesterol increased in the control trial postprandially. This response may negatively impact high and low density lipoprotein metabolism, leading to rapid catabolism of the former and enzymatic oxidation of the latter and appearance of small dense LDL particles to exacerbate oxidative stress [21]. Compared to previous findings, the lack of exercise effect may appear somewhat unexpected but is perhaps connected to the recreationally active training status of our volunteers. Enhanced physical fitness appears to influence the clearance of lipids as trained volunteers demonstrate lower postprandial profiles than sedentary counterparts [20]. This may account, in part, for the apparent differences in postprandial responses with studies with similar test meals. Trained volunteers may have an improved ability to metabolize lipids rapidly, conserving other nutrients or, alternatively, may benefit from training adaptations to LPL activity and hepatic very low density lipoprotein (VLDL)-TG secretion [20, 55].

At this time, the preceding exercise intensity remains a topic of debate. A collection of recent studies have reported that high intensity is effective in attenuating TG AUC [56, 57]. Further reports outline that high intensity intermittent exercise enhances postprandial fat oxidation rates and attenuates postprandial TG concentrations to a greater extent than moderate exercise [22, 57, 58]. However, this is not a unanimous finding as Tucker et al. [59] reported postprandial TG was unaffected by high intensity exercise, again dependent upon methodological variability. The same appears consistent for oxidative stress, as oxidative damage is attenuated by high intensity exercise [56]. Conversely, Canale et al. [51] reported preceding exercise of both moderate and high intensities, had no effects on oxidative stress and antioxidant status in healthy, active men. It appears that postprandial biomarkers appear extremely variable, dependent upon exercise intensity and methodological continuity (e.g. exercise and meal timing). Lopes Krüger et al. [60] further demonstrated this point by reporting TG incremental area under the curve responded more effectively to heavy intense exercise, while moderate exercise attenuated oxidative damage and improved vascular function.

This randomised control trial is not without limitations. Firstly, the absence of stringent dietary control in the days prior to the experimental conditions may be a limitation. Peddie and colleagues [21] report meals of differing composition affect PHTG clearance capacity, with greater lipid concentrations, for example, posing a sustained challenge. Intensive monitoring of dietary consumption could prevent volunteers consuming heterogeneous meals in the approaching days. However, we are confident this variable did not influence, given negligible differences between conditions in baseline metabolic and antioxidant biomarkers. On a related point and in retrospect, failing to quantify energy expenditure is also a limitation and might have provided deeper insight into its influence on the biomarkers. Moreover, despite sample size calculations, the study may be underpowered to detect significant interaction effects between experimental trials. Recruiting free-living volunteers for this type of intervention is perhaps the most frequent barrier encountered but will be an almost unavoidable challenge for this type of biomedical research. In addition, the effects of PHTG, and especially exercise, persist for several hours after the initial exposure so including further postprandial measures (beyond 6 h) and possibly into the following days, may provide deeper mechanistic scrutiny and understanding of this paradigm. Future research should attempt to explore the potential utility of high intensity exercise and its impact on DNA damage and associated biochemical parameters. As we investigated ESR as an indirect approach to characterise inflammation, attempts to directly catalogue postprandial cytokine activity and the subsequent downstream nature of the response, should also receive increased attention, as well as the interaction of inflammatory and oxidative signalling pathways, given both are often cited as disease risk factors. Finally, prospective studies should consider assessing the impact of the reported DNA damage with further molecular insights, quantifying the possible impact of this response, and if DNA repair processes are affected, especially following exercise.

## Conclusion

In summary, our findings indicate, for the first time, PHTG-induced oxidative stress characterised by DNA damage and lipid peroxidation. Antioxidant compounds increased postprandially though failed to prevent oxidative damage. In this instance, a single bout of moderate intensity walking could not mitigate the damaging effects of a HFM. Given the preliminary nature of our findings, the long-term mechanistic and clinical consequences, as well as the impact of different exercise doses requires further considered investigation, as the prevalence and availability of HFMs continues to rise.

## Data Availability

The datasets collected and analysed in the present study are available from the corresponding author on reasonable request.

## Abbreviations

^•^OH: Hydroxyl radical
AUC: Area under the curve
DNA: Deoxyribonucleic acid
ELISA: Enzyme-linked immunosorbent assay
EPR: Electron paramagnetic resonance
ESR: Erythrocyte sedimentation rate
H_2_O_2_: Hydrogen peroxide
HFM: High fat meal
HPLC: High performance liquid chromatography
IL-: Interleukin-
LDL: Low density lipoprotein
LOO^•^: Lipid peroxyl radical
LOOH: Lipid hydroperoxide
LPL: Lipoprotein lipase
NO^•^: Nitric oxide
O_2_^•–^: Superoxide
ONOO^−^: Peroxynitrite
PCA: Pulse contour analysis
PHTG: Postprandial hypertriglyceridemia
RBP-4: Retinol-binding protein-4
ROS: Reactive oxygen species
SSB: Single-strand breaks
TG: Triglycerides
TNF-α: Tumour necrosis factor-alpha
TRL: Triglyceride-rich lipoproteins
V̇O_2max_: maximal oxygen consumption
